# Patterns of Tumor Progression Predict Small and Tissue-Specific Tumor-Originating Niches

**DOI:** 10.3389/fonc.2018.00668

**Published:** 2019-01-10

**Authors:** Thomas Buder, Andreas Deutsch, Barbara Klink, Anja Voss-Böhme

**Affiliations:** ^1^Center for Information Services and High Performance Computing, Technische Universität Dresden, Dresden, Germany; ^2^Faculty of Informatics/Mathematics, HTW Dresden–University of Applied Sciences, Dresden, Germany; ^3^Institute for Clinical Genetics, Faculty of Medicine Carl Gustav Carus, Technische Universität Dresden, Dresden, Germany; ^4^German Cancer Consortium (DKTK), Dresden, Germany; ^5^German Cancer Research Center (DKFZ), Heidelberg, Germany; ^6^Center for Molecular Tumor Diagnostics (CMTD), National Center for Tumor Diseases (NCT), Dresden, Germany

**Keywords:** tumor formation and progression, cell-based stochastic model, cancer development, spatial Moran model, tumor origin

## Abstract

The development of cancer is a multistep process in which cells increase in malignancy through progressive alterations. Such altered cells compete with wild-type cells and have to establish within a tissue in order to induce tumor formation. The range of this competition and the tumor-originating cell type which acquires the first alteration is unknown for most human tissues, mainly because the involved processes are hardly observable, aggravating an understanding of early tumor development. On the tissue scale, one observes different progression types, namely with and without detectable benign precursor stages. Human epidemiological data on the ratios of the two progression types exhibit large differences between cancers. The idea of this study is to utilize data of the ratios of progression types in human cancers to estimate the homeostatic range of competition in human tissues. This homeostatic competition range can be interpreted as necessary numbers of altered cells to induce tumor formation on the tissue scale. For this purpose, we develop a cell-based stochastic model which is calibrated with newly-interpreted human epidemiological data. We find that the number of tumor cells which inevitably leads to later tumor formation is surprisingly small compared to the overall tumor and largely depends on the human tissue type. This result points toward the existence of a tissue-specific tumor-originating niche in which the fate of tumor development is decided early and long before a tumor becomes detectable. Moreover, our results suggest that the fixation of tumor cells in the tumor-originating niche triggers new processes which accelerate tumor growth after normal tissue homeostasis is voided. Our estimate for the human colon agrees well with the size of the stem cell niche in colonic crypts. For other tissues, our results might aid to identify the tumor-originating cell type. For instance, data on primary and secondary glioblastoma suggest that the tumors originate from a cell type competing in a range of 300 – 1,900 cells.

## Introduction

Cancer development is a multistep process in which cells acquire a certain number of progressive epigenetic and genetic alterations (1). This multistep process can be divided into a neutral and a selection phase. In the neutral phase, the epigenetic and genetic alterations do not confer a proliferative fitness advantage to the tumor precursor cells whereas cells gain such an advantage in the selection phase (2, 3). A single genetically altered cell does not necessarily induce tumor formation but is rather exposed to competition with its corresponding wild-type cells (4, 5). Tumor-originating cell refers to the wild-type cell of a certain type that acquires the first alteration in the multistep process of cancer development. Within the neutral phase, the progeny of the tumor-originating cell competes with wild-type cells within normal tissue homeostasis. Because this competition is controlled by the original tissue organization, the range of this competition is determined by the tissue structure which provides natural spatial boundaries for the spread of the progeny of the tumor-originating cell (6, 7). In order to induce tumor formation, the progeny of the tumor-originating cell has to establish within the tissue. This establishment is achieved by clonal expansion to a sufficiently large cell population (8). For some tissues, there is experimental evidence that this establishment is characterized by an outcompetition of wild-type cells within the homeostatic range of competition, e.g., the human colon (9).

However, in other tissues this phase of tumor development on the cellular scale is less understood. The main reason is a lack of knowledge regarding the tumor-originating cell type. Similar to the colon, it has been shown that stem cells within the hematopoietic system represent the tumor-originating cell type (10, 11). Further tumor-originating cell types in human tissues have been identified in the breast and prostate. In detail, luminal progenitor cells and basel progenitor cells can serve as tumor-originating cells in basal-like breast cancer (12) and prostate cancer (13), respectively. There is also evidence that non-stem cells can be the tumor-originating cell type, e.g., in oligodendroglioma (14). In other tissues, transplantation and tracing studies in mice revealed potential candidates for tumor-originating cells e.g., in glioblastoma, pancreatic, and basal cell cancers. Due to the limited applicability of these results obtained in mice experiments to human cancers, further studies are needed to obtain definitive evidence which of these cell types can serve as tumor-originating cells (15, 16).

On the tissue scale, tumors can progress sequentially, i.e., with a clinically detectable benign precursor stage. Alternatively, they can also progress by tunneling without such a prior benign precursor stage. Human epidemiological data allow to infer the progression patterns with respect to the ratios of tunneling vs. sequential progression of different tumors. Interestingly, these progression patterns differ largely between tissues although the underlying cellular multistep process is similar in essential characteristics. One observes that some tumors exhibit predominantly sequential progression, e.g., benign adenoma almost always develop prior to adenocarcinoma in the colon (9).

In contrast, glioblastoma develops in 90% of all cases without evidence of a less malignant precursor lesion (primary glioblastoma) and progresses in 10% of all cases from low-grade tumors (secondary glioblastoma) (17).

The idea of this study is to estimate the homeostatic range of competition of the tumor-originating cell type which sheds light on the hardly observable cellular scale of early cancer development. In detail, these estimates can be interpreted as number of altered cells within human tissues which are needed to induce tumor formation on the tissue scale. For this purpose, we utilize a Moran model with mutations (18–20) to describe cellular competition between wild-type cells and tumor cells. The Moran model is a cell-based mathematical model which is widely used to analyse the evolution of finite cell populations (21). We incorporate human epidemiological data on the progression patterns of cancers in order to estimate the homeostatic range of competition within human tissues corresponding to the number of cells which will inevitably induce tumor formation. Interestingly, our estimates are considerably small, tissue-specific and far away from the overall number of cells in a clinically observable tumor. We therefore propose that the fate of tumor development is decided in tissue-specific tumor-originating niches. This proposal is supported by our estimate of the tumor-originating niche size for the human colon which agrees well with the size of the stem cell niche in colonic crypts. In particular, we propose that a tumor-originating niche size of 300 – 1900 cells within the human brain can explain the ratio of primary and secondary glioblastoma.

## Materials and Methods

###  State Space and Representation of Benign and Malignant Tumor Subtypes

The multistep process in which cancer cells increase gradually in malignancy differs with respect to the number of steps, e.g., two steps in retinoblastoma (22) compared to seven steps in colon cancer (23). In our cell-based model, we only regard the last step within the neutral phase and the first step within the selection phase such that we obtain a two-step process. This coarse-grained approach is appropriate for our purpose since we are only interested in modeling tumor progression patterns and not quantities which are largely influenced by the precise number of steps, e.g., the time-scale of tumor development or intra-tumor heterogeneity. In the cellular two-step process, genetic or epigenetic alterations can transform wild-type cells into benign tumor cells which can further progress to malignant tumor cells. We assume that the benign progeny of the tumor-originating cell competes with wild-type cells and can clonally expand within normal tissue homeostasis. The parameter *N* in our model describes the homeostatic range of this competition. We further assume that monoclonal conversion of wild-type cells into benign tumor cells within the homeostatic range of competition *N* represents the establishment of benign tumor cells within a tissue. In contrast, if a benign tumor cell progresses to a malignant tumor cell we identify this occurrence with fixation in the homeostatic range of competition because of the high fitness advantage of malignant cells (19). Once benign or malignant tumor cells fixated, a benign or malignant tumor, respectively, will inevitably be detected either directly if *N* is sufficiently large or at a later time due to an altered growth behavior destroying tissue homeostasis after fixation. Notice that the timescale between fixation and detection potentially ranges from zero to several years. In the model, a further progression from benign fixation to malignant tumor detection or after a possible benign tumor detection is neglected. These assumptions are motivated by experimental observations within the colon where mutant cells either go extinct or fixate in the colonic stem cell niche (24). In other tissues, much less is known about the relation between tumor initiation and detection which motivates our study.

**Figure 1:**
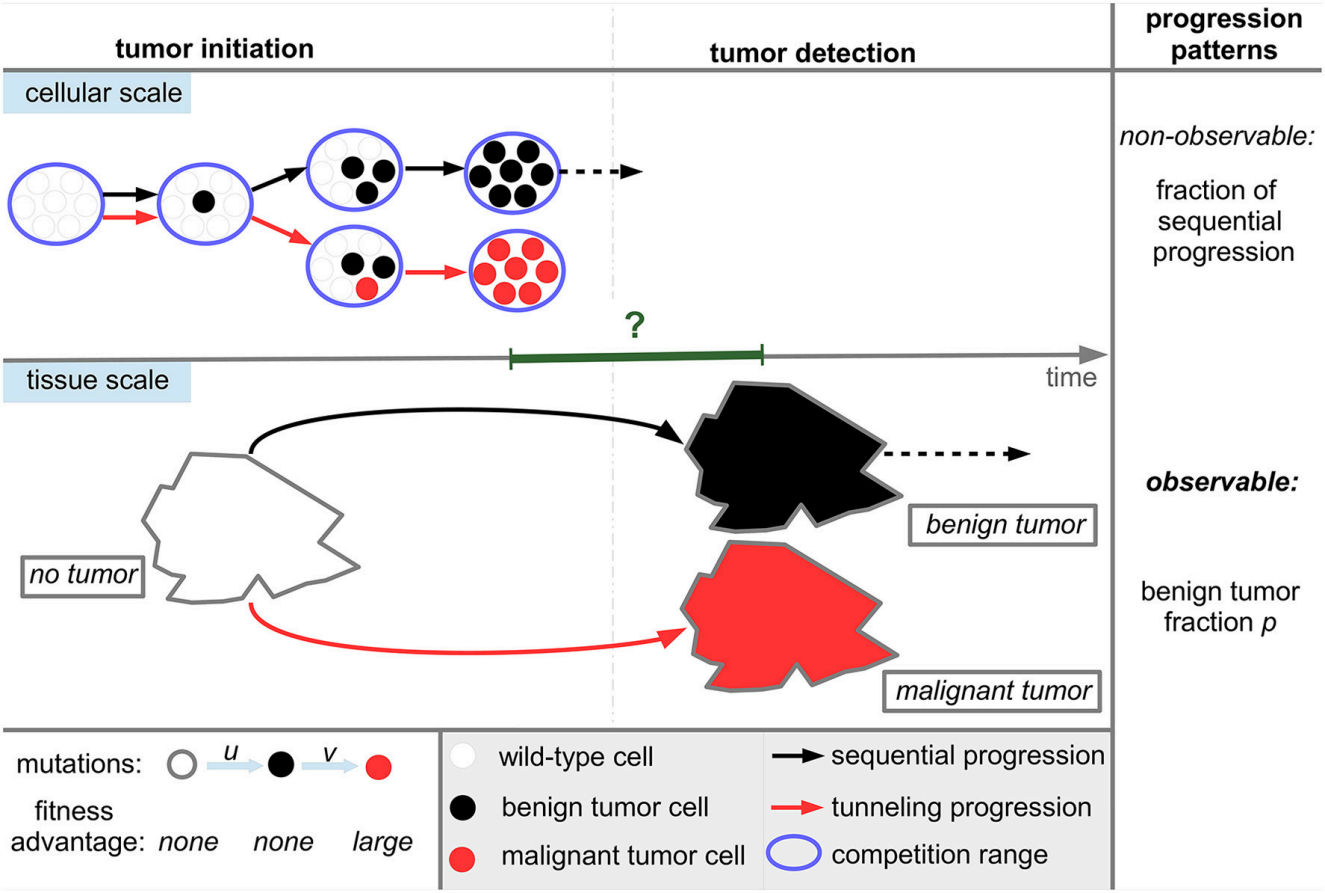
Tumor progression types and patterns in the model. Wild-type cells can progress to benign tumor cells during proliferation with mutation probability *u* and further progress to malignant tumor cells with probability *v*. Wild-type and benign tumor cells neutrally compete with each other within the homeostatic range of competition which is modeled by Moran dynamics, see Figure 2. We assume that tumor cells establish within the tissue if they clonally expand to fixation in the homeostatic range of competition corresponding to the parameter *N* in the model. Then, a tumor will inevitably be detected either directly if *N* is sufficiently large or at a later time due to an altered growth behavior destroying tissue homeostasis after fixation. Correspondingly, the timescale between fixation and detection, indicated by the green interval, potentially ranges from zero to several years. The cellular dynamics lead to two distinct progression types at the tissue scale, namely sequential progression and tunneling progression. The benign tumor fraction *p* determines the progression pattern. A further progression from benign fixation to malignant tumor detection (dotted line in the cellular scale) or after a possible benign tumor detection (dotted line in the tissue scale) is neglected.

The state space of the underlying stochastic process of the model is *S* = {0, 1, 2, …., *N, E*} where states 0 to *N* represent the occurrence of the respective number of benign tumor cells without the occurrence of malignant tumor cells. State *E* indicates the presence of a malignant tumor cell. States *N* and *E* correspond to later emergence of benign and malignant tumor subtypes and therefore to sequential and tunneling tumor progression, see also Figure 1. Both states *N* and *E* are absorbing states of the underlying stochastic process, see also Text S1 for details.

### Dynamics in the Model

**Figure 2:**
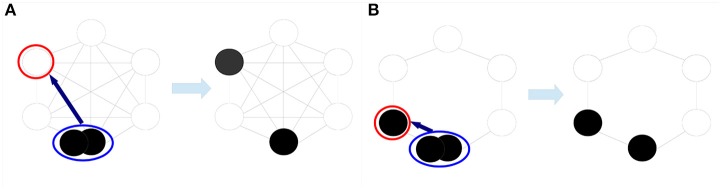
Moran dynamics with different spatial cell arrangements. In the Moran dynamics, a randomly chosen cell proliferates (blue circle) and replaces a neighboring cell which undergoes cell death (red circle). In **(A)**, the space-free dynamics is illustrated, i.e., each cell can be replaced by any other cell. In **(B)**, only neighboring cells can be replaced representing a one-dimensional cell arrangement.

In order to describe competition between cells and tumor cell progression, we adopt a Moran model with mutations. This model class has mostly been investigated from a theoretical point of view (19, 25, 26). Recently, we applied a Moran model to evaluate tumor regression in pilocytic astrocytoma (20). Moran models are appropriate to describe a population of fixed size *N* which represents the homeostatic range of competition in our model. The dynamics is as follows. One cell is randomly chosen to undergo cell death and is replaced by the offspring of another chosen cell, see also Figure 2. During proliferation, a genetic or epigenetic alteration can lead to tumor cell progression. Wild-type cells can progress to benign tumor cells with probability *u* and benign tumor cells progress to malignant tumor cells with probability *v*. We assume that initially all cells are wild-type cells. Hence, the process starts in state 0.

### Analysis of the Model

#### Choice of Spatial Cell Arrangement

Theoretical studies demonstrated that the interplay between tissue structure, the population size *N* and mutation probabilities *u* and *v* in Moran models are crucial for the dynamics of the model (19, 26, 27). In particular, it has been shown that the absorption probability in state *N* on regular structures is the highest if all cells can potentially compete with each other and the lowest for a one-dimensional cell arrangement (19). Since the tumor-originating cell type is unknown for most cancers also the spatial cell arrangement and realization of competition is unknown (4, 28). Therefore, we consider a space-free and a one-dimensional cell arrangement in order account for this uncertainty by deriving a lower and an upper bound for the absorption probabilities. Figure 2 illustrates the Moran dynamics on these two structures. For the precise definition of the underlying stochastic processes, see Text S1.

#### Tumor Progression Patterns in the Model

Three parameter regimes within the model can be distinguished with respect to the tumor progression patterns. Within the *sequential fixation* regime, the benign tumor cell population is primarily able to reach size *N* before a benign tumor cell progresses to a malignant tumor cell. This regime corresponds to primarily sequential progression on the tissue scale. In the *tunneling* regime (25) a malignant clone will occur before the benign population is able to reach size *N* which corresponds to primarily tunneling progression in the model. In the *borderline* regime (27) both sequential fixation and tunneling are possible corresponding to both progression types on the tissue scale. An asymptotic classification of the model behavior with respect to these parameter regimes for large *N* has been theoretically derived in a space-free model (29) and in lattice-like cell arrangements (26). We showed in a previous work that the exact progression pattern described by the absorption probability in state *N* in the space-free model solely depends on the so-called risk coefficient $\gamma :=N\sqrt{v}$ (20). Analogously, we derive here that the absorption probability in the one-dimensional model solely depends on a one-dimensional risk coefficient $\gamma_{1D}=N\sqrt[{3}]{v}$.

For technical details regarding the choice of the parameter regime for the model analysis and the precise derivation of the absorption probabilities of the underlying stochastic processes, see Text S1, Table S2 and Figure S5.

## Results

### The Homeostatic Range of Competition in Human Tissues Is Surprisingly Small

**Figure 3:**
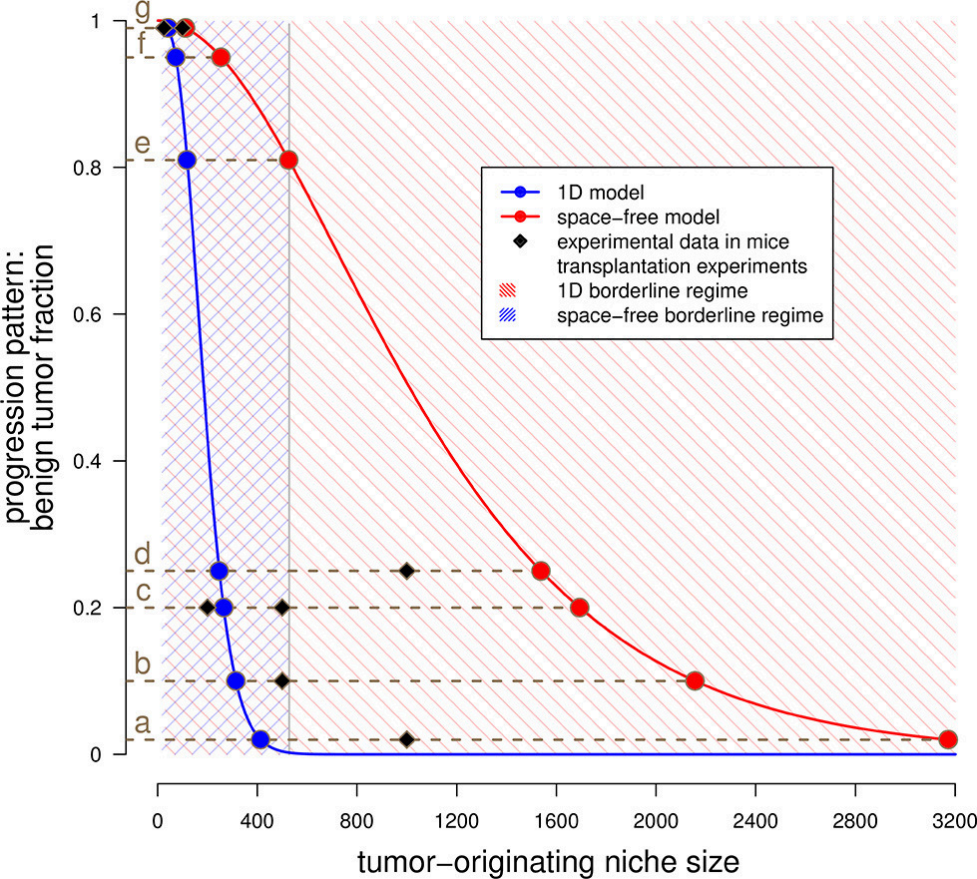
Estimated tumor-originating niche sizes based on tumor progression patterns. This plot shows the benign tumor fraction in the space-free (red) and one-dimensional (blue) model as function of the tumor-originating niche size. The blue curve has been numerically evaluated, see Text S1, equation (12). The red curve represents the plot of equation (3) in Text S1. The shaded areas illustrate the regimes in which both sequential and tunneling progression are possible for the space-free and the 1*D* model, see Table 1. The dots indicate the estimated tumor-originating niche size, (a)–(g) indicate different tissues and the squares represent experimental data for these tissues, see Table 2.

Our analysis allows to determine the progression patterns in both the space-free and the one-dimensional model in dependency of the competition range *N*. Interestingly, we find that a considerably small value of *N* corresponds to primarily tunneling progression in both the space-free and one-dimensional model. In detail, we find that a homeostatic range of competition in human tissues >4,530 cells implies that primarily malignant tumors develop no matter the spatial cell arrangement within the tissue. Moreover, the estimates of the parameter *N* largely depend on the considered underlying spatial cell arrangement. In particular, the smaller the number of neighboring cells, the smaller is the estimated competition range. The estimated values for a mutation probability *v* = 10^−6^ per cell division (30) are summarized in Table 1 and visualized in Figure 3. Note that these conclusions also hold for other values of *v* although a smaller value of *v* would increase and a larger value of *v* would decrease the estimates, see Tables S3 and S4.

**Table 1:** Homeostatic range of competition and corresponding tumor progression patterns.

**Spatial model**	**Space-free model**	**Progression patterns**	**Tumor fate**
*N* ≤ 17	*N* ≤ 29	Primarily sequential	Only benign
17 < *N* ≤ 528	29 < *N* ≤ 4530	Sequential and tunneling	Benign and malignant
*N*>528	*N*>4530	Primarily tunneling	Only malignant

*The table provides a classification of the progression patterns in human tissues in dependency of the homeostatic range of competition N and the spatial cell arrangement. Interestingly, these estimates imply that a homeostatic competition range >4,530 cells implies that only malignant tumors and no benign tumors develop. For these estimates, we have chosen a mutation rate from benign to malignant cells of v = 10^−6^. Primarily sequential and primarily tunneling progression patterns refer to a fraction of 99.9% of sequential and tunneling progression, respectively*.

### Fate of Tumor Development Is Decided in Small Tissue-Specific Tumor-Originating Niches

Our model allows to estimate the range of cellular competition *N* in different human tissues. For these estimations, we calibrate the space-free and 1*D* model with epidemiological data on the diagnosed fraction of benign and malignant tumor subtypes. We performed an extensive literature research to obtain these data which allow to estimate the risk coefficients $\gamma =N\sqrt{v}$ and $\gamma_{1D}=N\sqrt[{3}]{v}$ in the following way. We equal the clinically diagnosed fraction of benign tumors *p* with the absorption probabilities of the underlying stochastic processes. Subsequently, we numerically calculate the risk coefficients by evaluating the inverse of the absorption probability function at the diagnosed fraction of benign tumors *p*, i.e., γ = α^−1^(*p*). The resulting estimates of the competition ranges in various tissues are provided in Table 2 and visualized in Figure 3. Our model predicts that the range of competition is considerably small compared to the overall number of cells in a tumor. Note that we do not assume any upper bound for the parameter *N* in our model. Moreover, although the estimates are considerably small, the range of competition largely depends on the tissue. For example, the estimated competition range within the liver is 383–2,837 cells whereas the estimates for the bone marrow are 18–31 cells.

**Table 2:** Estimation of the homeostatic competition range *N* in different tissues.

**Epidemiological data**					**Model predictions**		**Mice data**
	**Tissue**	**Benign precursor**	**Malignant tumor**	**Benign fraction *p***	***N*_space-free_**	***N*_1_*_D_***	**Needed cells for tumor formation in mice**
a)	Liver	Hepatocellular adenoma	Hepatocellular carcinoma	2% (31)	2,837	383	1,000 (32)
b)	Brain	Low-grade astrocytoma	Glioblastoma	10% (17)	1,928	291	500 (33)
c)	Breast	Ductal carcinoma *in situ*	Invasive ductal carcinoma	20% (34)	1,514	246	200 (35) – 500 (36)
d)	Skin	Nevus	Melanoma	25% (37)	1,375	230	1, 000 (38)
e)	Stomach	Gastric adenomas	Gastric cancer	81% (39)	471	111	200 (40)
f)	Meninges	Benign meningioma	Aggressive meningioma	95% (41)	227	67	NA
g)	Colon	Colonic adenoma	Adenocarcinoma	99% (42)	100	39	25 (43) – 100 (44)
h)	Bone marrow	MGUS	Myeloma	99.9^*^% (45)	31	18	NA

*This table summarizes human epidemiological data on benign and malignant tumor subtypes. We calibrate the model such that the absorption probabilities in state N given by equations (3, 12) in Text S1 are equal to the diagnosed benign tumor fraction p. We assume a mutation probability from benign to malignant cells of v = 10^−6^ throughout these calculations. The last column contains the necessary number of injected cells to obtain a tumor in mice transplantation experiments. The estimates and mice data are also illustrated in Figure 3*.

* *, estimated, MGUS, monoclonal gammopathy of undetermined significance*.

The tumor-originating cell within the human colon has been identified to be almost always a stem cell with a first hit in the APC gene, and a second hit in this gene is sufficient to induce adenoma formation, a benign precursor of malignant adenocarcinoma. These stem cells reside at the bottom of so-called niches within colonic crypts and are capable of self-renewal and multilineage differentiation (9). It has been demonstrated that tumor-originating cells neutrally compete with wild-type stem cells for a position within the spatially restricted stem cell niche (24). Either such an altered stem cell goes extinct due to this competition or eventually replaces all wild-type stem cells within the stem cell niche. This process has been termed monoclonal conversion and represents almost always the first step of tumor formation within the human colon (9). Hence, the monoclonal conversion of the stem cell niche by the progeny of the tumor-originating cell with loss of the APC gene induces the establishment of an adenoma on the tissue scale. Importantly, the estimate of the tumor-originating niche size for the human colon agrees well with the stem cell niche size in colonic crypts of about 40 cells (46) but surely <100 cells (47).

Overall, these results can be interpreted as existence of a tissue-specific tumor-originating niche in which the fate of tumor development is decided long before a tumor becomes detectable. The small estimates suggest that the fixation of tumor cells within the tumor-originating niches trigger new processes which accelerate the expansion of tumor cells and destroy normal tissue homeostasis. Indeed, it has been shown that the fixation of mutant cells within the colonic stem cell niche induces a higher rate of crypt fission which accelerates the spread of mutated cells (48). Based on our results, we propose that a tumor-originating niche size of 291–1,928 cells within the human brain might be responsible for the clinically observed fraction of primary and secondary glioblastoma, see Table 2).

### Predicted Tumor-Originating Niche Sizes in Human Tissues Agree Well With Mice Injection Experiments

We compare the estimated tumor-originating niche sizes for human tissues in Table 2 with available data of tumor initiation experiments in mice from the literature. Obviously, such data are not available in human tissues which is one main motivation for our modeling approach. Interestingly, it turns out that our estimates correspond very well to the necessary cell numbers for tumor induction in mice experiments (32, 33, 35, 36, 38, 40, 43, 44), see also Figure 3. This observation supports the existence of tumor-originating niches by showing that a critical number of malignant tumor cells is necessary for tumor development and that this number can either be reached by clonal expansion within the tumor-originating niche or directly by injection of a sufficient large number of malignant tumor cells.

## Discussion

On the tissue scale, one observes tumor progression types with and without detectable benign precursor stages. Data on the progression patterns with respect to the ratios of these progression types exhibit large differences between tissues. The underlying cellular processes causing these progression patterns are hardly observable and remain unclear. In this work, we shed light on the cellular multistep process of tumor development on the cellular scale by estimating the homeostatic competition range of the tumor-originating cell type in several human tissues. Our model is based on competition between wild-type and tumor cells and assumes that a sufficient amount of tumor cells is needed for tumor formation. We estimate this number by fitting the model to human data on the diagnosed ratios of benign and malignant tumor subtypes. Our model predicts that this number is considerably small compared to the overall number of cells in a clinically detectable tumor and largely depends on the tissue which can be interpreted as existence of a tissue-specific tumor-originating niche. Hence, our results suggest that the fate of tumor development is decided long before a tumor becomes detectable. This finding implies that the fixation of tumor cells within the tumor-originating niche might trigger additional mechanisms that accelerate tumor development after normal tissue homeostasis is voided. Our model is based on several simplifying assumptions. We assume that benign tumors develop from neutrally evolving tumor cells. This is not always the case, e.g., for Barrett's Esophagus, a precursor to esophageal adenocarcinoma, it has been shown that there is positive selection already in the benign phase. However, recently it has been claimed that benign tumor development is characterized by neutral evolution for many cancer types (2). Here, to estimate the niche sizes, we only rely on data which was derived for cancer types with neutral evolution in the benign phase. Moreover, the data about the diagnosed fraction of benign tumors which we utilize for model calibration is only a lower bound for the portion of benign tumor cell fixation. First, a certain fraction of tumors could potentially progress after benign cells fixate within the competition range in the subsequent phase until tumor detection. In this case, a malignant tumor develops instead of a benign one which means that the true benign tumor fraction is smaller than the portion of benign tumor cell fixation. On the other hand, the data themselves are biased since the clinical detection of a benign tumor depends on many factors, such as its size and accessibility. Therefore, the benign tumor fractions reported in the literature could be smaller than the true benign tumor fraction since there might be non-detected benign tumors. However, an underestimation of the portion of benign tumor fixation implies that the predicted tumor-originating niche sizes are overestimated which means that our main finding of small and tissue-specific tumor-originating niche sizes is even more pronounced.

Interestingly, our estimates of the tumor-originating niche size of about 39 cells for colon cancer agrees well with the number of stem cells found in one colonic crypt (46). Indeed, it is the current understanding that colon adenomas and carcinomas develop within one colonic crypt with intestine stem cells likely to be the cell type of origin (49). This demonstrates that our model might be utilized to predict tumor-originating niche sizes, thereby allowing to infer the potential cell type of tumor-origin for cancers from other tissues in which the origin is still under debate, e.g., for glioblastoma (50). Glioblastoma can be divided into two classes dependent on the progression dynamics. In about 90% of cases, glioblastoma occur *de novo*, i.e. without evidence of a less malignant precursor lesion (primary glioblastoma) whereas 10% develop slowly by progression from low-grade gliomas (secondary glioblastoma). Using this data, our model predicts that the size of the tumor-originating niche from which glioblastoma develop is about 291 – 1,928 cells. Neural stem cells (NSCs) of the subependymal zone (SEZ) have been suggested as a potential cell of origin for glioblastoma. Moreover, recent experimental evidence regarding NSCs in the SEZ of the adult brain suggests that the total number and fate of NSCs is regulated by a density-dependent mechanism (51). Importantly, the finding in (51) that the fate of a NSC, e.g., activation or quiescence, is coupled to its neighbors perfectly fits to our hypothesis of cells competing within a certain range. Interestingly, the authors also suggest that the fate of active NSCs is coupled to the total number of neighboring NSCs in a shared locally restricted area which suggests that this area is a potential candidate for the tumor-initiating niche in the adult brain. It would be interesting to investigate if the range of coupled NSCs fits to our predicted size of the tumor-originating niche for glioblastoma. In human basal-like breast cancers luminal cells have been identified as tumor-originating cell type. Our study suggests that these cells are organized such that they compete with each other within a range of 246 to 1514 cells and that the fate of the tumor is already decided if there is an accumulation of a corresponding number of altered cells. This finding can guide further experimental studies. In a similar manner, our estimates might help to identify candidates for tumor-originating cell types in human tissues which is a first important step toward the development of future targeted therapies.

The potential existence of tumor-originating niches in which tumor fate is decided at an early stage of the cellular multistep process supports the view that cancer development is an ecological process (52, 53). Ecology studies the dynamics of communities of species and their interactions and describes the origin of new species. From this point of view, the size of the tumor-originating niche might represent a critical effective population size that has to be reached by the progeny of the tumor-originating cell type in order to establish a tumor on the tissue scale. A deeper understanding of the processes and the origin of the tumor-originating niche contributes to the understanding of the early phase of tumor development. In this work, we demonstrated how observable quantities on the tissue scale might be utilized to achieve this goal.

## Author Contributions

TB wrote the paper together with AD, BK and AV-B. AD, BK and AV-B supervised the study and gave substantial input to the manuscript. TB conceived, designed and analyzed the model together with AV-B.

### Conflict of Interest Statement

The authors declare that the research was conducted in the absence of any commercial or financial relationships that could be construed as a potential conflict of interest.
