# Supplementary material for: Patterns of Tumor Progression Predict Small and Tissue-Specific Tumor-Originating Niches
Source: Front Oncol. 2019 Jan 10;8:668. doi: 10.3389/fonc.2018.00668 (PMC6335293; doi:10.3389/fonc.2018.00668)
Supplement: Supplementary file 1 [file Data_Sheet_1.PDF]

Supplementary Material

S1 Text

The space-free model.

The space-free model presented in the main text is a three-type $\textsc{Moran}$ model with mutation probabilities u, v, $0 \leqslant u, v < 1$ in which the offspring of a cell can replace any other cell. Formally, the model is a $\textsc{Markov}$ process $(X_t)_{t\geqslant 0}$ on the state space $S = \{0, 1, 2, ..., N, E\}$ where $1, \ldots, N$ represent the number of benign cells in the population when no malignant cell is present and state E represents the existence of a malignant tumor cell. The dynamics is determined by the rate matrix $Q = (q(k,l))_{k,l\in S}$ with

$$q(k,l) = \begin{cases} Nu, & k = 0, l = 1, \\ \frac{(N-k)k}{N}(1-v) + \frac{(N-k)(N-k-1)u}{N}, & 1 \leqslant k \leqslant N-1, l = k+1, \\ \frac{(N-k)k(1-u)}{N}, & 1 \leqslant k \leqslant N-1, l = k-1, \\ kv, & 1 \leqslant k \leqslant N-1, l = E, \\ -\sum\limits_{\substack{m\in S \\ m\neq k}} q(k,m), & l = k, \\ 0, & \text{else.} \end{cases} \tag{1}$$

The rate $q(k, k+1)$ for an increase of the state is composed as follows. There are two possibilities for such an increase. First, the offspring of a benign cell can replace a wild-type cell. Since there are $N-k$ wild-type cells within the population, the rate to choose one of them is $N-k$. The probability that a benign cell is selected for reproduction is $\frac{k}{N}$ and the offspring does not undergo a mutation with probability $1-v$. This yields a rate of $(N-k)\cdot\frac{k}{N}\cdot(1-v)$. Second, the state can also increase by a reproduction of a benign cell with subsequent mutation of the offspring which replaces a wild-type cell. The rate for this event is given by $(N-k)u\cdot\frac{N-k-1}{N}$. The sum of the rates for both possibilities yields the entry in (1) and the other rates are similarly obtained.
The states N and E are absorbing states of the process since $q(N,l) = q(E,l) = 0$ for $l \in S$. By assuming

$$Nu \ll 1, \tag{2}$$

i.e. that mutations to benign cells are rare such that each arising benign cell mutant can be investigated independently [1], we derived in [2] the absorption probability of this process in state N. This derivation utilizes first step analysis in order to obtain a linear system of equations for the absorption probabilities starting with $k, 1 \leqslant k \leqslant N$, type-I cells. Subsequently, $\textsc{Cramer's}$ rule allows to derive the particular absorption probability starting with one type-I mutant. We obtained an exact solution for finite values of N and also an asymptotic result by taking the limit for $N \to \infty$ which reads

$$\alpha(\gamma) = \frac{1}{I_0(2\sqrt{\gamma})}, \tag{3}$$

where $\gamma := N\sqrt{v}$ and $I_n, n \in \mathbb{N}_0$, denote the modified BESSEL functions of the first kind, see [3]. For the rigorous derivation of this result, see the Supplementary Material in [2].

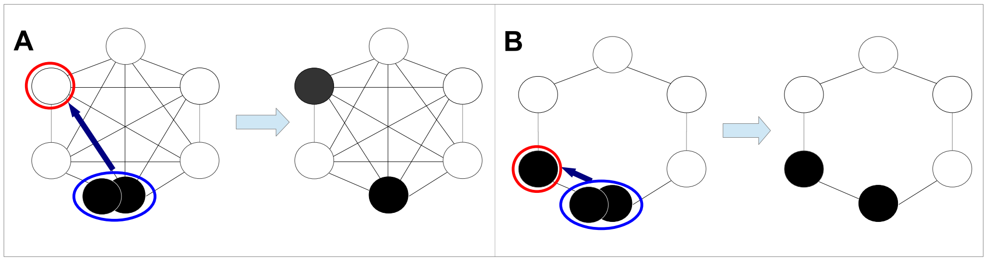

Moran dynamics with different spatial cell arrangements. In the MORAN dynamics, a randomly chosen cell proliferates (blue circle) and replaces a neighboring cell which undergoes cell death (red circle). In **A**, the space-free dynamics is illustrated, i.e. each cell can be replaced by any other cell. In **B**, only neighboring cells can be replaced representing a one-dimensional cell arrangement. (Reprint of Fig. 2 in the main text)

The one-dimensional model.

The one-dimensional model presented in the main text is a spatial three-type MORAN model with mutation probabilities u, v, $0 \leqslant u, v < 1$ in which each cell can only be replaced by the offspring of the two neighboring cells. For the analysis, we assume that

$$Nu \ll \sqrt[3]{v} \tag{4}$$

and define the 1D risk coefficient γ_{1D} as

$$N\sqrt[3]{v} =: \gamma_{1D}. \tag{5}$$

Analogously to (2), assumption (4) guarantees that mutations to benign cells are rare such that each arising benign cell mutant can be investigated independently, see [1]. Hence, for the analysis we can neglect mutations to benign cells if the system is already in a state $k > 0$. We only consider the last benign mutant which eventually leads to absorption in states N or E. Therefore, we study a modified process with $u = 0$ conditioned that this benign clone will not go extinct. Note that if there are any benign cells in the system, then there is always exactly one connected benign cell population.

Formally, this modified one-dimensional model is a MARKOV process on the state space $S = \{1, 2, 3, ..., N, E\}$, where $1, \ldots, N$ represent the number of benign cells in the population when no malignant cell is present and state E represents the existence of a malignant tumor cell, and

rate matrix $Q = (q(k,l))_{k,l\in S}$ which reads

$$q(k,l) = \begin{cases} 1-v, & 1 \leqslant k \leqslant N-1, l = k+1, \\ 1, & 2 \leqslant k \leqslant N-1, l = k-1, \\ kv, & 1 \leqslant k \leqslant N-1, l = E, \\ -\sum\limits_{\substack{m\in\widetilde{S} \\ m\neq k}} q(k,m), & l = k, \\ 0, & \text{else}. \end{cases} \tag{6}$$

The rate $q(k, k+1)$ for an increase of the state is composed as follows. For an increase of the state, the offspring of a benign cell must replace a wild-type cell.

- If $k = 1$, i.e. there is only one benign cell in the system, then exactly this cell has to be selected to reproduce. The neighboring cell that is replaced is always a wild-type cell. However, during reproduction, there must not be a mutation which implies a rate of $1-v$.
- If $k > 1$, then only the two benign cells at the boundary of the connected benign cell population have to be selected for reproduction since otherwise the offspring would replace a benign cell. One of them is selected with rate 2. The chosen cell has two neighbors, a benign and a malignant cell. Thus, the wild-type cell is chosen to be replaced with probability $\frac{1}{2}$. Finally, the offspring of the benign cell must not undergo a mutation which holds with a probability of $1-v$. Hence, the offspring of a benign cell replaces a wild-type cell with rate $2 \cdot \frac{1}{2} \cdot (1-v) = 1-v$.

The other rates in (6) are similarly obtained. The states N and E are absorbing states of the process since $q(N,l) = q(E,l) = 0$ for $l \in S$. Note that a discrete-time version of this process has been first introduced in [4].

Derivation of the absorption probabilites. For the rates (6) we set $q(k) := -q(k,k)$ and get

$$\begin{aligned} q(1) &= q(1,E) + q(1,2) = 1, \\ q(N) &= q(E) = 0, \\ q(k) &= q(k,k+1) + q(k,k-1) + q(k,E) = 2 + v(k-1), \quad 2 \leqslant k \leqslant N-1. \end{aligned}$$

We further regard the embedded $\textsc{Markov}$ chain with transition probabilities

$$p(i,j) = \begin{cases} \frac{q(i,j)}{q(i)}, & i \neq j, \\ 1, & i = j = E, \\ 1, & i = j = N, \\ 0, & \text{else}, \end{cases}$$

in which the entries unequal to 0 look as follows

$$\begin{aligned} p(1,E) &= v, \\ p(1,2) &= 1-v, \\ p(k,E) &= \frac{kv}{2+v(k-1)}, \quad 2 \leqslant k \leqslant N-1, \\ p(k,k+1) &= \frac{1-v}{2+v(k-1)}, \quad 2 \leqslant k \leqslant N-1, \\ p(k,k-1) &= \frac{1}{2+v(k-1)}, \quad 2 \leqslant k \leqslant N-1, \\ p(N,N) &= p(E,E) = 1. \end{aligned}$$

The absorption probabilities for the underlying stochastic process with transition matrix $P = \left(p_{i,j}\right)_{i,j \in S}$ is obtained as follows. Denote by $\alpha_{\mathsf{1D}}^N = \left(\alpha_{\mathsf{1D}}^N(i,v)\right)_{i \in S}$ the absorption probabilities where $\alpha_{\mathsf{1D}}^N(i,v)$ describes the absorption probability in state N starting from state i.
First step analysis yields

$$\alpha_{\mathsf{1D}}^N(i,v) = \sum_{j \in S} p(i,j)\alpha_{\mathsf{1D}}^N(j,v), \quad i \in S.$$

It holds that $\alpha_{\mathsf{1D}}^N(E,v) = 0$, $\alpha_{\mathsf{1D}}^N(N,v) = 1$ and therefore

$$\begin{aligned} \alpha_{\mathsf{1D}}^N(i,v) &= \sum_{j=1}^{N} p(i,j)\alpha_{\mathsf{1D}}^N(j,v) \\ &= \sum_{j=1}^{N-1} p(i,j)\alpha_{\mathsf{1D}}^N(j,v) + p(i,N) \\ &= \begin{cases} (1-v)\alpha_{\mathsf{1D}}^N(2,v), & i=1 \\ \frac{1}{2+v(i-1)}\alpha_{\mathsf{1D}}^N(i-1,v) + \frac{1-v}{2+v(i-1)}\alpha_{\mathsf{1D}}^N(i+1,v), & 2 \leqslant i \leqslant N-2, \\ \frac{1}{2+v(N-2)}\alpha_{\mathsf{1D}}^N(N-2,v) + \frac{1-v}{2+v(N-2)}, & i = N-1. \end{cases} \end{aligned}$$

Hence,

$$\begin{aligned} -\alpha_{\mathsf{1D}}^N(1,v) + (1-v)\alpha_{\mathsf{1D}}^N(2,v) &= 0 \\ \frac{1}{2+v(i-1)}\alpha_{\mathsf{1D}}^N(i-1,v) - \alpha_{\mathsf{1D}}^N(i,v) + \frac{1-v}{2+v(i-1)}\alpha_{\mathsf{1D}}^N(i+1,v) &= 0, \quad 2 \leqslant i \leqslant N-2, \\ \frac{1}{2+v(N-2)}\alpha_{\mathsf{1D}}^N(N-2,v) - \alpha_{\mathsf{1D}}^N(N-1,v) &= -\frac{1-v}{2+v(N-2)}. \end{aligned}$$

By multiplying each equation with the corresponding denominator, one gets an equivalent system $P_N^{'}\widetilde{\alpha}_{1\text{D}}^{N} = b$ for a $(N-1)\times(N-1)$ matrix $P_N^{'}$ and $\widetilde{\alpha}_{1\text{D}}^{N} := \left(\alpha_{1\text{D}}^{N}(i,v)\right)_{i=1,\dots,N-1}$. This system reads in tableau form as follows.

$$
\begin{array}{c|cccccc|c}
 & \alpha_{1\text{D}}^{N}(1,v) & \alpha_{1\text{D}}^{N}(2,v) & \alpha_{1\text{D}}^{N}(3,v) & \dots & \dots & \alpha_{1\text{D}}^{N}(N-1,v) & 1 \\
\hline
1 & -1 & 1-v & 0 & 0 & \cdots & 0 & 0 \\
2 & 1 & -2-v & 1-v & 0 & \ddots & \vdots & \vdots \\
3 & 0 & 1 & -2-2v & 1-v & \ddots & \vdots & \vdots \\
\vdots & \vdots & \ddots & \ddots & \ddots & \ddots & 0 & \vdots \\
\vdots & \vdots & \ddots & \ddots & \ddots & \ddots & 1-v & 0 \\
N-1 & 0 & \cdots & \cdots & 0 & 1 & -2-(N-2)v & -(1-v)
\end{array}
\tag{7}
$$

CRAMER'S rule can be applied to derive the absorption probability in state N starting with a single type-1 cell, i.e.

$$\alpha_{1\text{D}}^{N}(1,v) = \frac{\det \widetilde{P}_N^{'}}{\det P_N^{'}},$$

where $\widetilde{P}_N^{'}$ is the matrix formed by replacing the first column of $P_N^{'}$ by the column vector b. By applying LAPLACE expansion along the last column of $P_N^{'}$ we obtain

$$
\begin{aligned}
\det P_N^{'} &= (-1)^{2N-2}(-2-(N-2)v)\det P_{N-1}^{'} \\
&\quad + (-1)^{2N-3}(1-v)\begin{vmatrix}
-1 & 1-v & 0 & 0 & \cdots & 0 \\
1 & -2-v & 1-v & 0 & \ddots & \vdots \\
0 & 1 & -2-2v & 1-v & \ddots & \vdots \\
\vdots & \ddots & \ddots & \ddots & \ddots & 0 \\
\vdots & \ddots & 0 & 1 & -2-(N-4)v & 1-v \\
0 & \cdots & \cdots & \cdots & 0 & 1
\end{vmatrix} \\
&= (-2-(N-2)v)\det P_{N-1}^{'} - (1-v)\cdot(-1)^{2N-4}\cdot 1\cdot \det P_{N-2}^{'} \\
&= (-2-(N-2)v)\det P_{N-1}^{'} - (1-v)\det P_{N-2}^{'},
\end{aligned}
$$

where the determinant in the first equality was evaluated by applying LAPLACE expansion along the last row. The result of these calculations is a second order difference equation with non-constant coefficients which reads as follows.

$$
\begin{aligned}
&\det P_N^{'} = (-2-(N-2)v)\det P_{N-1}^{'} - (1-v)\det P_{N-2}^{'}, \quad N \geqslant 4, \\
&\det P_3^{'} = 2v+1, \\
&\det P_2^{'} = -1.
\end{aligned}
\tag{8}
$$

Since the mutation rate v can be considered close to zero we can assume that $(1-v)$ is close to one and obtain the modified equation

$$D_N = (-2-(N-2)v)D_{N-1} - D_{N-2}, \quad D_3 = 2v+1, D_2 = -1, \quad N \geqslant 4. \tag{9}$$

This modified difference equation can be solved and the solution can be simplified with MATHEMATICA [5] which yields

$$\begin{aligned} D_N = \frac{(-1)^{N+1}\pi}{v} \Bigg(& J_{N+\frac{2}{v}}\left(\frac{2}{v}\right)\left((2v+1)Y_{1+\frac{2}{v}}\left(\frac{2}{v}\right) - Y_{2+\frac{2}{v}}\left(\frac{2}{v}\right)\right) \\ & + Y_{N+\frac{2}{v}}\left(\frac{2}{v}\right)\left(J_{2+\frac{2}{v}}\left(\frac{2}{v}\right) - (2v+1)J_{1+\frac{2}{v}}\left(\frac{2}{v}\right)\right)\Bigg). \end{aligned} \tag{10}$$

Here, J denotes a BESSEL function of the first kind and Y denotes a BESSEL function of the second kind, see [3]. We will use the solution D_N of the modified difference equation as approximation for $\det P_N'$.
The determinant of $\widetilde{P}_N'$ is calculated as follows. The matrix $\widetilde{P}_N'$ is given by

$$\widetilde{P}_N' = \begin{pmatrix} 0 & 1-v & 0 & \cdots & \cdots & 0 \\ 0 & -2-2v & 1-v & 0 & \ddots & 0 \\ 0 & 1 & -2-2v & 1-v & \ddots & \vdots \\ \vdots & \ddots & \ddots & \ddots & \ddots & \vdots \\ 0 & \ddots & \ddots & \ddots & \ddots & 1-v \\ -(1-v) & 0 & \cdots & \cdots & 1 & -2-(N-2)v \end{pmatrix}.$$

Therefore, the determinant can be calculated by applying LAPLACE expansion along the first column and evaluating the determinant of the remaining triangular matrix, i.e.

$$\begin{aligned} \det \widetilde{P}_N' &= (-1)^N \cdot (-(1-v)) \begin{vmatrix} 1-v & 0 & \cdots & \cdots & \cdots & 0 \\ -2-2v & 1-v & 0 & \ddots & \ddots & 0 \\ 1 & 2-2v & 1-v & \ddots & \ddots & \vdots \\ 0 & \ddots & \ddots & \ddots & \ddots & \vdots \\ \vdots & \ddots & \ddots & \ddots & \ddots & 0 \\ 0 & \cdots & \ddots & \ddots & \ddots & 1-v \end{vmatrix} \\ &= (-1)^{N+1} \cdot (1-v)^{N-1}. \end{aligned} \tag{11}$$

Using this result and the approximation derived in equation (10) allows the approximation of

the absorption probability $\alpha_{\mathsf{1D}}^{N}(1,v)$ with CRAMER'S rule

$$\alpha_{\mathsf{1D}}^{N}(1,v) \approx \frac{\det \widetilde{P}_N'}{D_N} =$$

$$\frac{v(1-v)^{N-1}}{\pi\left(J_{N+\frac{2}{v}}\left(\frac{2}{v}\right)\left((2v+1)Y_{1+\frac{2}{v}}\left(\frac{2}{v}\right)-Y_{2+\frac{2}{v}}\left(\frac{2}{v}\right)\right)+Y_{N+\frac{2}{v}}\left(\frac{2}{v}\right)\left(J_{2+\frac{2}{v}}\left(\frac{2}{v}\right)-(2v+1)J_{1+\frac{2}{v}}\left(\frac{2}{v}\right)\right)\right)}. \tag{12}$$

S2 Table

Comparison of the approximate solution and simulation results of the 1D model.

N	v	simulated absorption prob. $\widehat{\alpha}^N(1,v)$	$\alpha^N(1,v)$	deviation
10	0.0015	0.7931	0.8226	0.0295
10	0.001	0.8541	0.8761	0.0220
10	0.0001	0.9837	0.9864	0.0027
10	0.00001	0.9984	0.9986	0.0002
10	0.000001	0.9998	0.9999	0.0001
10	0.0000001	1	1	0
50	0.0015	0.0004	0.0004	0
50	0.001	0.0019	0.0022	0.0003
50	0.0001	0.2428	0.2568	0.0140
50	0.00001	0.8216	0.8299	0.0083
50	0.000001	0.9795	0.9806	0.0011
50	0.0000001	0.9979	0.9980	0.0001
100	0.001	0	0	0
100	0.0001	0.0040	0.0044	0.0004
100	0.00001	0.3025	0.3105	0.0080
100	0.000001	0.8530	0.8567	0.0037
100	0.0000001	0.9836	0.9840	0.0004
100	0.00000001	0.9983	0.9984	0.0001
500	0.0001	0	0	0
500	0.00001	0	0	0
500	0.000001	0.0019	0.0019	0
500	0.0000001	0.2429	0.2444	0.0015
500	0.00000005	0.4389	0.4407	0.0018
750	0.000001	0	0	0
750	0.0000001	0.0385	0.0388	0.0003
750	0.00000005	0.1276	0.1283	0.0007
750	0.00000001	0.5531	0.5542	0.0011
1000	0.000001	0	0	0
1000	0.0000001	0.0040	0.0041	0.0001
1000	0.00000005	0.0266	0.0268	0.0002
1000	0.00000001	0.3025	0.3034	0.0009

Comparison of the analytical approximation and simulation results from 10000 trajectories of the underlying stochastic process of the one-dimensional model for the probability of absorption in state N.

S3 Table

Tumor progression patterns in dependency of the risk coefficients.

progression pattern	space-free model	1D model
primarily sequential	$\gamma \leqslant 0.031$	$\gamma_{1D} \leqslant 0.187$
borderline	$0.031 < \gamma < 4.53$	$0.187 < \gamma_{1D} < 5.281$
primarily tunneling	$\gamma \geqslant 4.53$	$\gamma_{1D} \geqslant 5.281$

This table summarizes the risk coefficient regimes with respect to the different progression patterns of the model. Primarily sequential and primarily tunneling progression patterns refer to a fraction of 99.9% of sequential and tunneling progression, respectively.

S4 Table

The influence of the mutation probability on the model estimates.

progression pattern	v	space-free model	1D model
primarily sequential	10^{-5}	$N \leqslant 10$	$N \leqslant 8$
	10^{-6}	$N \leqslant 29$	$N \leqslant 17$
	10^{-7}	$N \leqslant 100$	$N \leqslant 39$
both sequential and tunneling	10^{-5}	$10 < N \leqslant 1408$	$8 < N \leqslant 245$
	10^{-6}	$29 < N \leqslant 4530$	$17 < N \leqslant 528$
	10^{-7}	$100 < N \leqslant 14080$	$40 < N \leqslant 1138$
primarily tunneling	10^{-5}	$N > 1408$	$N > 245$
	10^{-6}	$N > 4530$	$N > 528$
	10^{-7}	$N > 14080$	$N > 1138$

This table summarizes regimes for the parameter N with respect to the different progression patterns of the models in dependency of the mutation probability v. Primarily sequential and primarily tunneling progression patterns refer to a fraction of 99.9% of sequential and tunneling progression, respectively.

S5 Figure

The absorption probability in the 1D model in dependency of γ_{1D}.

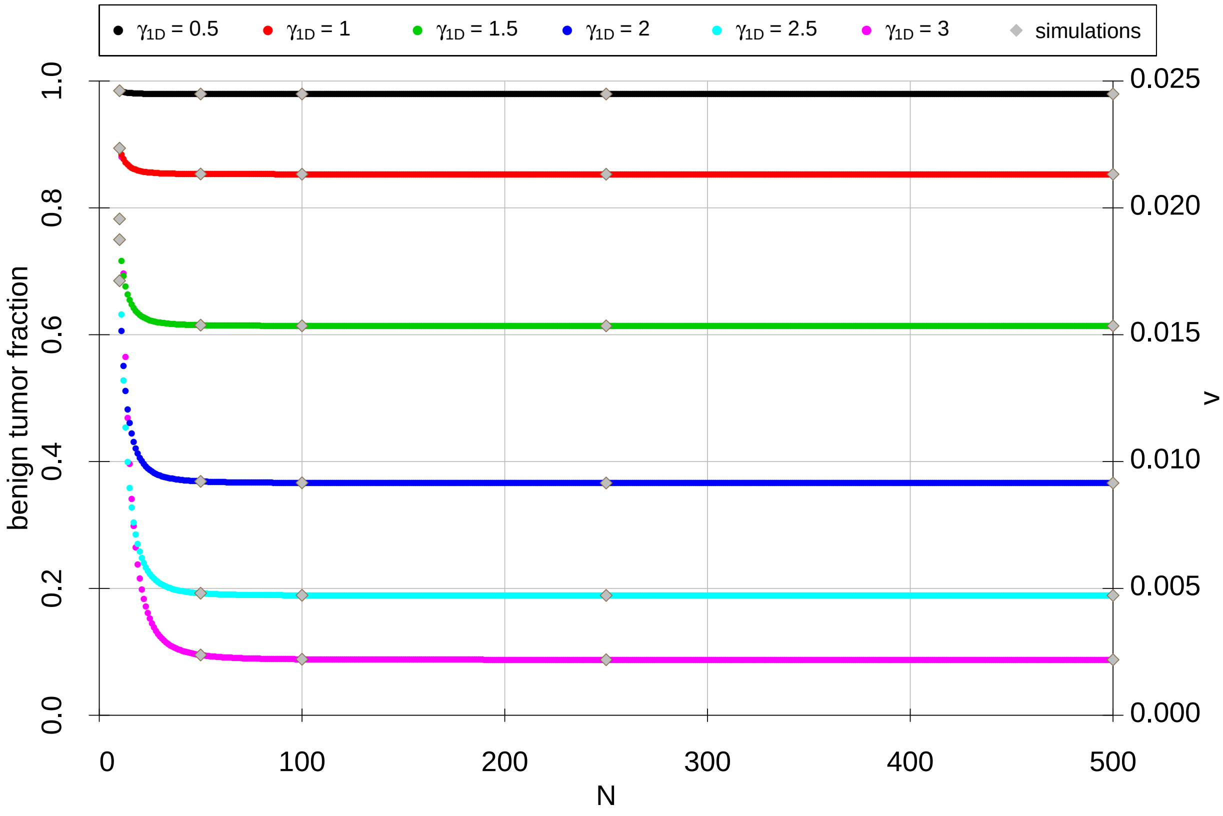

We numerically approximated the absorption probability in state N for different values of N and v such that the risk coefficient γ_{1D} is constant. This analysis suggests that the absorption probability solely depends on the risk coefficient γ_{1D} for approximately $N \geqslant 40$. The squares indicate the results of simulation studies of the absorption probability in state N and therefore the benign tumor fraction in the model.

References

[1] R. Durrett and S. Moseley, "Spatial moran models i. stochastic tunneling in the neutral case," *The Annals of Applied Probability*, vol. 25, no. 1, p. 104, 2015.

[2] T. Buder, A. Deutsch, B. Klink, and A. Voss-Böhme, "Model-based evaluation of spontaneous tumor regression in pilocytic astrocytoma," *PLoS computational biology*, vol. 11, no. 12, p. e1004662, 2015.

[3] M. Abramowitz, *Handbook of Mathematical Functions, With Formulas, Graphs, and Mathematical Tables,*. Dover Publications, Incorporated, 1974.

[4] N. L. Komarova, “Spatial stochastic models for cancer initiation and progression,” *Bulletin of Mathematical Biology*, vol. 68, no. 7, pp. 1573–1599, 2006.

[5] W. R. Inc., “Mathematica, Version 11.3.” Champaign, IL, 2018.
